# Interoception and Obsessive-Compulsive Disorder: A Review of Current Evidence and Future Directions

**DOI:** 10.3389/fpsyt.2021.686482

**Published:** 2021-08-25

**Authors:** Laura B. Bragdon, Goi Khia Eng, Amanda Belanger, Katherine A. Collins, Emily R. Stern

**Affiliations:** ^1^Department of Psychiatry, New York University School of Medicine, New York, NY, United States; ^2^Nathan S. Kline Institute for Psychiatric Research, Orangeburg, NY, United States; ^3^Department of Psychiatry, Icahn School of Medicine at Mount Sinai, New York, NY, United States

**Keywords:** obsessive-compulsive disorder, interoception, sensory phenomena, disgust, evidence-based treatment

## Abstract

Disrupted interoceptive processes are present in a range of psychiatric conditions, and there is a small but growing body of research on the role of interoception in obsessive-compulsive disorder (OCD). In this review, we outline dimensions of interoception and review current literature on the processing of internal bodily sensations within OCD. Investigations in OCD utilizing objective measures of interoception are limited and results mixed, however, the subjective experience of internal bodily sensations appears to be atypical and relate to specific patterns of symptom dimensions. Further, neuroimaging investigations suggest that interoception is related to core features of OCD, particularly sensory phenomena and disgust. Interoception is discussed in the context of treatment by presenting an overview of existing interventions and suggesting how modifications aimed at better targeting interoceptive processes could serve to optimize outcomes. Interoception represents a promising direction for multi-method research in OCD, which we expect, will prove useful for improving current interventions and identifying new treatment targets.

## Introduction

OCD affects 1–3% of adults (1) and is associated with significant economic cost and a chronic course (2, 3). It is characterized by recurrent, intrusive thoughts, images, urges, and/or sensory-perceptual experiences that cause distress (obsessions) and repetitive behaviors performed to reduce this distress (compulsions). Clinical presentation and symptom content can vary greatly across individuals, with the most reliable dimensions including harm/checking, contamination/cleaning, and symmetry/ordering (4, 5). Though gold-standard treatments including serotonin reuptake inhibitors and cognitive behavioral therapy (CBT) work for many patients with OCD, a significant portion do not achieve meaningful symptom reduction (6, 7), which may be due to this heterogeneity.

Seminal conceptualizations of OCD that emphasize the role of cognitions and fear in the development and maintenance of symptoms are particularly relevant for obsessions and compulsions related to preventing or avoiding a feared outcome or bad event (e.g., “I check my stove because I am afraid it has been left on and will burn down my house”). Indeed, traditional anxiety-based models form the foundation for evidence-based CBT interventions such as exposure and response prevention ExRP; (8–11). However, these models do not account as well for those symptoms of OCD that are less fear-driven, including behaviors that are more motivated by sensory or visceral sensations such as “not-just-right” experiences (NJREs; “I need to arrange objects until they look just right”), disgust, and physical urges (“I feel dirty or sticky so I have to wash my hands repeatedly”). These types of symptoms—frequently referred to as “sensory phenomena” —are prominent in ~50–80% of patients with OCD (12, 13) and have been the topic of an emerging body of work that aims to understand the psychological and neural correlates of these symptoms (14, 15) in an attempt to identify more targeted treatments.

A growing body of research has begun to investigate the role of sensory processing in OCD, including the processing of internal, or interoceptive stimuli (and the focus of this paper) and external (exteroceptive) stimuli [See Grimaldi and Stern (16) and Collins et al. (17) for more information on exteroception in OCD]. Interoception, defined as the detection, integration, and interpretation of internal bodily signals (18, 19). Body sensations provide important information necessary to maintain homeostasis, influence attentional, and emotional processes, impact decision-making, and motivate behavior (18–23). Indeed, interoception is posited to be a core facet of emotional, behavioral, and cognitive regulation (20, 23–27). Therefore, it is perhaps not surprising that disrupted interoceptive processes are present in a wide range of psychiatric conditions, including anxiety, depression, addiction, psychosis, and anorexia (18, 19, 22, 28, 29). With regard to OCD, there are several antecedents to compulsions that, in addition to being fear-based, could be driven at least in part by altered processing of body signals (such as NJREs and disgust). There are additional aspects of altered interoception in OCD that may not necessarily drive compulsive behavior in the traditional sense, but nonetheless can negatively impact disease course and treatment response in the disorder (such as anxiety sensitivity). An investigation into the behavioral and neural correlates of interoception in OCD has the potential to improve personalization of treatment and identify novel targets for intervention.

In this review, we discuss the existing literature examining the role of interoception in OCD with the goal of highlighting its relevance to clinical heterogeneity and the optimization of treatment outcomes. Extending prior work (30), we first provide an overview of the different aspects of interoception and their neural bases before discussing current research on interoception and related constructs in OCD. Then, we consider interoception in the context of clinical intervention and discuss implications for research and treatment.

## Methods

A PubMed literature search was conducted in July 2021 to identify the existing investigations of interoception in OCD or OC symptoms using the MeSH terms “obsessive compulsive OR obsessions OR compulsions” AND “interoception OR interoceptive OR body awareness.” Reference lists of articles were also reviewed for additional relevant literature.

## Results

The PubMed search resulted in 169 publications, of which 3 examined interoceptive accuracy (31–33) and 1 investigated interoceptive sensibility (34) in OCD. Review of reference lists yielded 1 additional article examining interoceptive accuracy in OCD (35) and 1 additional article examining interoceptive sensibility and OCD symptoms in and undergraduate sample.

## Neural Basis of Interoception

The neurobiology underlying interoception has been fairly well-delineated. Ascending small-diameter primary fibers carry visceral (e.g., about heart, lungs, gastrointestinal, urogenital), somatic (e.g., muscles, joints, skin), and homeostatic information (e.g., about temperature, mechanical stress, cellular activity) from tissues in the body to brainstem nuclei e.g., parabrachial, nucleus of the solitary tract, periaqueductal gray (20, 21, 36). These afferents reach the thalamus and the hypothalamus (37), and primarily through the thalamus, project to other subcortical (insula, hippocampus, amygdala) and cortical (cingulate, somatosensory, orbitofrontal, and medial prefrontal) regions (20, 21, 38).

The insula is considered a hub in this network (20, 39–41) and has been implicated in a wide variety of interoceptive processes including disgust (42), substance craving (43, 44), pain (45), and physical urges (46, 47). Converging evidence from several neuroimaging studies identify a tripartite functional parcellation of the insula into posterior, ventral anterior, and dorsal anterior subdivisions. The posterior insula is involved in sensory processing and has functional connections to the sensorimotor regions including somatosensory cortex (postcentral gyrus) and primary and secondary motor areas (precentral gyrus, supplementary motor area) (20, 48–50). The ventral and dorsal subdivisions of the anterior insula have different functional connectivity profiles (48). The ventral anterior insula is functionally connected to the limbic and paralimbic regions and is involved in emotion processing (48, 51, 52), whereas the dorsal anterior insula is functionally connected to regions involved in cognitive control and salience detection (53–55) including the dorsal anterior cingulate (ACC) and lateral prefrontal cortex (48, 51, 56). It has been proposed that different aspects of interoception follow this tripartite division of function of the insula: afferents carrying sensory signals from the body are first represented at the posterior insula before relaying information to the anterior insula, where interoceptive signals are re-represented with greater complexity through the integration of emotional (ventral) and cognitive (dorsal) information transmitted from connecting cortical and sub-cortical regions (30, 39, 40, 57, 58).

## Dimensions of Interoception

Research has distinguished between separate facets of interoception, including the capacities to detect, discriminate, and evaluate the magnitude of different bodily signals [(59), see Tables 1, 2]. Garfinkel et al. (71), for instance, distinguished between the objective detection of bodily sensations (“interoceptive accuracy”), the subjective experience of bodily sensations (“interoceptive sensibility”) and the metacognitive awareness of interoceptive accuracy (e.g., whether a person believes they are accurately identifying bodily sensations, “interoceptive awareness”). Interoceptive accuracy and sensibility have been the focus of most research in healthy and clinical samples.

**Table 1:** Interoceptive dimensions.

**Interoceptive Component**	**Definition**	**Method of Assessment**	**Neural correlates**	**Findings in OCD**
Attention	Observation of internal body sensations	Focusing task:Attend to sensations in specific organ (e.g., Simmons et al. (60); Farb et al. (61)	Right dorsal middle anterior insula (60), posterior insula (61)	N/A
Detection	Presence/absence of conscious report	Example:Subjects judge whether external tones occur simultaneous to pulse/heartbeat (e.g., Khalsa et al. (62)	Anterior insula (63)	N/A
Magnitude	Intensity of body sensations	Example: Dial ratings of internal sensation intensity [e.g. Khalsa et al. (64)]	N/A	N/A
Discrimination	Localization of sensation to a specific system, and differentiation from other sensations	Organ specific ratings, heartbeat discrimination task [e.g., Aziz et al. (65); Khalsa et al. (64)]	Anterior cingulate (65)	N/A
Interoceptive accuracy (IAcc)	Objective accuracy of interoceptive states	Examples:Heartbeat detection Task: Comparison of subjective heartbeat count to actual heartbeats measured with EEG [e.g., Schandry et al. (66)]	Subcortical: Insula and right dorsal anterior insula in particular, midbrain, ventral striatumCortical anterior cingulate, orbitofronal somatosensory (41, 63, 67–69)	•Decreased IAcc:° Heartbeat counting task (32)° Muscle tension task (35) •Increased IAcc (70)
Interoceptive Awareness (IA)	Meta-cognitive awareness of interoceptive accuracy	Agreement between objective and subjective report: Subjective confidence ratings during heartbeat detection task compared to IAcc [e.g., Garfinkel et al. (71)]	N/A	N/A
Interoceptive Sensibility (IS)	Subjective assessment of how internal body signals are appraised, regulated, and impact behavior	Self-report, for example:MAIA (72), BPQ (73) BAQ (74) Confidence ratings	Anterior-mid insula, cingulate cortex, orbitofrontal cortex, somatosensory and sensorimotor regions (75–78)	•Compared to healthy controls, OCD demonstrated higher noticing, distracting, worrying, emotional awareness, listening but lower trusting on MAIA •Higher noticing related to responsibility/harm,symmetry/ordering symptoms •Higher distracting related to unacceptable/taboo thoughts (34)
Emotional Evaluation of Interoceptive Signals (IE*)*	Emotional appraisal of internal bodily signals	MAIA Not Worrying Subscale (72), ASI Physical Subscale (79)	Posterior, dorsal, and anterior insula, dorsal anterior cingulate (75)	•OCD appraises internal physical sensations more negatively than controls (34, 80) •Negative appraisal of internal sensations correlated with responsibility/harm, contamination/washing, symmetry/ordering, certainty/doubting (34, 81, 82)

*ASI, Anxiety Sensitivity Index; BPQ, Body Perception Questionnaire; BAQ, Body Awareness Questionnaire; EEG, electroencephalogram; MAIA, Multidimensional Assessment of Interoceptive Awareness; OCD, obsessive-compulsive disorder*.

**Table 2:** Reliability and validity of select interoceptive assessments.

**Interoceptive Dimension**	**Assessment**	**Reliability**	**Correlations Between Interoceptive Constructs**		
			**Interoceptive Awareness (IA)**	**Interoceptive Sensibility (IS)**	**Emotional Evaluation of Interoceptive Signals (IE*)***
Interoceptive accuracy (IAcc)	Heartbeat detection (counting) task	Retest reliability (2 months) = 0.60 (83) α = 0.89 (84)	*r* = 0.16, *p* = 0.17 (85)	MAIA (84): Noticing *r* = −0.05, *ns* Not Distracting *r* = −0.06, *ns* Not Worrying *r* = 0.08, *ns* Attention Regulation*r* = 0.20, *p = 0.02* Emotional Awareness*r* = −0.05, *ns* Self-Regulation *r* = −0.03, *ns* Body Listening *r* = 0.00, *ns* Trusting *r* = 0.08, *ns* BPQ: *r* = 0.06, *ns* (71) BAQ: *r* = 0.18, *p* < 0.05 (86) Confidence rating: *r* = 0.28, *p* < 0.05 (71)	MAIA Not Worrying:*r* = 0.08, *ns* (84)
Interoceptive Awareness (IA)	Agreement between objective and subjective report: Subjective confidence ratings during heartbeat detection task compared to IAc			Confidence rating: *r* = −0.02, *p* = 0.84 (85)	
Interoceptive Sensibility (IS)	Self-report, e.g., MAIA, BPQ, BAQ, confidence ratings	MAIA (72),α = 0.66–0.87 (72), retest reliability (M days= 113, SD = 4.3)= 0.66–0.79 (87) BPQ (73) BPQ-SF ω = 0.83–91, retest reliability (1 week) = 0.91–0.96 (88), Confidence ratings			
Emotional Evaluation of Interoceptive Signals (IE*)*	Self-report, e.g., MAIA Not Worrying subscale, ASI Physical subscale	MAIA Not Worrying:α = 0.67 (72); retest reliability (*M* days= 113, SD = 4.3)= 0.76 (87) ASI Physical:α = 0.76–0.89 (89); ASI-3 retest reliability (3 months) = 0.70 (90)^a^			

*Samples are non-clinical unless otherwise noted*.

*MAIA, Multidimensional Assessment of Interoceptive Awareness; BPQ, Body Perception Questionnaire; BPQ-SF, Body Perception Questionnaire-Short Form; BAQ, Body Awareness Questionnaire; ASI, Anxiety Sensitivity Index; ASI-3, Anxiety Sensitivity Index-3 α, Cronbach's alpha (internal consistency); ω, Categorical Omega (internal consistency); r, Pearson correlation coefficient*.

a *Sample of treatment seeking smokers*.

Interoceptive accuracy (IAcc) reflects the objective perceptual accuracy of interoceptive states. It is most commonly measured by a heartbeat detection task where individuals are asked to count the number of heartbeats occurring over a period of time, which is then compared to the actual number of heartbeats measured with pulse plethysmography (PPG) or electrocardiogram (ECG). IAcc has been found to be associated with emotional processing (91–93). Neuroimaging studies of heartbeat detection have linked individual differences in the functioning of several regions including the midbrain, ventral striatum, anterior cingulate cortex, somatosensory cortex, and the insula to greater interoceptive accuracy [e.g., (41, 63, 67, 94)]. From among these areas, the right dorsal anterior insula appears to be the most reliably positively associated with interoceptive accuracy across studies (41, 63, 95). It has been proposed that this insular subregion may contribute to instantaneous subjective feelings from the body that generate a sense of the present moment (40, 67).

Interoceptive sensibility (IS) relies on self-report and represents the subjective assessment of how internal body signals are appraised, regulated, and impact behavior (72, 73, 96, 97), and is frequently assessed using self-report questionnaires. Interoceptive sensibility is arguably the broadest of the three dimensions, encompassing several different aspects of subjective body processing. Commonly used scales include the Body Perception Questionnaire BPQ; (73), Body Awareness Questionnaire [BAQ; (74)], and the Multidimensional Assessment of Interoceptive Awareness [MAIA; (72)]. While both the BPQ and BAQ measure an individual's general tendency to notice and be aware of their body sensations, the MAIA includes 8 subscales assessing different cognitive, emotional, and behavioral aspects and was designed to differentiate between “adaptive” and “maladaptive” forms of IS (98). Furthermore, the *emotional evaluation of interoceptive signals* [IE; (99, 100)] is a subcomponent of IS typically measured via self-report questions characterizing how an individual emotionally interprets bodily sensations. For example, IE is assessed by items in the MAIA “Not Worrying” subscale (e.g., “I start to worry that something is wrong if I feel any discomfort”). Existing literature suggests that IAcc and IS are often unrelated and emphasizes the utility in distinguishing between these two dimensions (30, 75). For example, IE has been shown to be unrelated to IAcc and more representative of top-down processing (99). See Table 2 for correlations among measures of interoceptive dimensions.

A number of neuroimaging studies have investigated IS, the majority of which utilized a single dimension measure such as the BPQ. Critchley et al. (41) for instance, found an association between self-reported awareness of body sensation assessed with the BPQ and gray matter volume in the insula. In a large sample of healthy adults, Wang et al. (76) found that IS was negatively correlated with functional connectivity between three pairs of brain regions: ventral anterior insula and superior temporal gyrus, dorsal anterior cingulate cortex and middle frontal cortex, and amygdala and medioventral occipital cortex. In clinical samples, IS has been linked to connectivity between the ACC and orbitofrontal cortex (OFC) (77) as well as between anterior insula and somatosensory regions (78). Only one study has examined the neural correlates of IS using a multidimensional measure. Using a dimensional reduction approach on the MAIA in a healthy sample, Stern et al. (75) reported a 3-factor solution, of which, one factor corresponded to reduced ability to regulate attention to body sensation, greater tendency to distract from uncomfortable body sensation, and greater worrying over body sensation. Scores on this component were related to increased BOLD activity in the anterior-mid insula, along with the cingulate cortex, and somatosensory/sensorimotor regions during interoceptive attention focusing. Compared to IAcc, the brain regions implicated in IS are less clear given fewer number of studies and differences in measures (i.e., MAIA vs. BPQ) and modalities (i.e., gray matter volume vs. functional connectivity). Whereas, literature most consistently implicates the involvement of the dorsal anterior insula in IAcc, findings in IS are less consistent. However, there does appear to be some overlap between neural correlates of these two facets, including the anterior cingulate (75, 76) and somatosensory areas (75).

## Interoceptive Dimensions and OCD

Few studies have directly examined interoceptive dimensions in OCD utilizing the measures discussed above. Interoceptive accuracy has been examined in two studies using the heartbeat detection task with mixed results. Yoris et al. (70) reported increased accuracy in OCD patients compared to controls when counting heartbeats, whereas Schultchen et al. (32) and DeMartini et al. (31) found decreased accuracy (31, 32, 70). It is possible that variation in counting procedure [e.g., tapping hand in Yoris et al. (70) vs. silent counting in Schultchen et al. (32) and DeMartini et al. (31)] contributed to inconsistent findings. Though heartbeat detection tasks are the most commonly used measure of IAcc, the role of the cardiovascular system is not as clearly relevant for OCD as it is for other psychiatric conditions like panic disorder. In a study utilizing a different approach to measuring the processing of internal sensation in OCD, Lazarov et al. (35) used a muscle tension task and found that individuals with OCD were less accurate than healthy controls and individuals diagnosed with other anxiety disorders in their ability to produce specific muscle tensions when feedback was not given (35). Although these few studies represent important first steps investigating interoceptive accuracy in OCD, given the limited number of studies, task variability, and mixed findings, more research is needed before conclusions can be drawn. It has been recommended that tasks assess IAcc across organ different systems (e.g., cardiovascular, respiratory, gastrointestinal, etc.) to create a more reliable and comprehensive “interoceptive profile” (59). Indeed, future investigations may benefit from including multisystem tasks to clarify IAcc in OCD.

Our group has examined interoceptive sensibility in OCD utilizing the MAIA (34). In our investigation, compared to healthy controls, individuals with OCD reported hyperawareness of bodily sensations. Further, the OCD group demonstrated a more maladaptive profile of IS including increased distraction from and worry about uncomfortable sensations. Within OCD, different dimensions of IS also related to clinical heterogeneity. For example, increased tendency to notice bodily sensations correlated with higher severity of symmetry/ordering (e.g., “feelings that something is not just right and behaviors designed to achieve order, symmetry, or balance”) and responsibility for harm (e.g., “thoughts and behaviors related to harm and disasters”) symptoms. Greater worry about body sensations, which corresponds to the IE sub-dimension of interoceptive sensibility, was related to increased severity of both the responsibility for harm and contamination symptom dimensions. In an undergraduate sample, Jokić and Purić (101) found that a similar pattern of MAIA subscales demonstrated significant (albeit small) correlations with overall OC symptoms (101). Therefore, existing findings suggest that obsessive-compulsive symptoms may relate to a profile of interoceptive sensibility characterized by awareness of bodily sensations, reliance on sensations for information, appraisal of sensations as threatening, and the tendency to respond to aversive sensations with cognitive avoidance.

Anxiety sensitivity (AS) is a construct related to interoceptive sensibility that has been more frequently researched in OCD patients. Anxiety sensitivity reflects fear of anxiety-related body sensations (79). AS is most commonly assessed utilizing self-report methods, specifically by the 16-item Anxiety Sensitivity Index (ASI), which allows calculation of a total score in addition to subscale scores measuring fear of social evaluation (ASI-Social), cognitive (ASI-Cognitive), and physical symptoms ASI-Physical; e.g., “When my stomach is upset, I worry that I might be seriously ill” (102). Greater fear of physical anxiety symptoms, as measured by ASI-physical, is correlated with increased worrying about body sensations and a greater tendency to regulate emotional states through attention to body sensation as measured by the MAIA (72), and thus corresponds to the interoceptive sensibility subconstruct of *emotional evaluation of interoceptive signals*. AS is considered to be a transdiagnostic construct and is known to be broadly related to a range of psychopathology including panic disorder, posttraumatic stress disorder (PTSD), generalized anxiety disorder (GAD), substance-use disorders, and suicidal ideation (103–107). OCD samples demonstrate higher levels of physical anxiety sensitivity compared to healthy controls and comparable levels to anxiety disorder samples (80). Greater fear of physical symptoms is not only associated with overall OCD symptom severity, but also has been related to increased contamination, symmetry/ordering, and certainty/doubting symptoms (81, 82, 108). A recent longitudinal study in adolescents found a bidirectional association between AS and OC symptoms suggesting that not only is AS a risk factor for developing OC symptoms, but experiencing symptoms also increases the prospective risk of elevated AS over a 2-year period (109).

To the best of our knowledge, no study has yet evaluated AS in individuals with OCD using neuroimaging but existing literature on AS in individuals and subthreshold anxiety symptoms seems to consistently implicate the anterior insula. Higher ASI-total score was associated with neural activity in the insula (posterior and dorsal anterior) and dorsal anterior cingulate (dACC) in individuals with panic disorder with agoraphobia when viewing fearful and angry emotional faces (110). Additionally, dorsal anterior insula and amygdala activity was found to be higher among individuals with subthreshold anxiety symptoms in a face viewing task (111). Using the 36-item Anxiety Sensitivity Index (Revised; ASI-R) in a sample of individuals with panic disorder, Kim et al. (112) reported that total ASI-R score was associated with greater functional anisotropy (i.e., indicator of white matter integrity) in the white matter regions near the insula, corpus callosum, posterior limb, retrolenticular parts of the internal capsule, posterior thalamic radiata, posterior corona radiata, and sagittal striatum (112). These white matter findings were consistent with a previous report stating that AS is associated with functional connectivity between the insula and other neural regions (including the thalamus and amygdala) that are known to modulate interoceptive processing (113–115).

Of the three dimensions proposed by Garfinkel et al. (71, 96), interoceptive sensibility, or the self-reported assessment of interoceptive ability, appears to be most consistently abnormal in OCD. Specifically, studies using the MAIA and ASI-physical subscale indicate that OC symptoms may relate to an attentiveness to internal sensations and a reliance on sensations to clarify emotional states and inform behavior (34, 101). Further, and perhaps most consistent with the subdimension of emotional evaluation of interoceptive signals, studies demonstrate that individuals with OCD appraise internal bodily sensations as threatening and respond to aversive sensations with cognitive avoidance (34, 80, 101). These findings are somewhat consistent with CBT models, which emphasize the role of appraisal and avoidance in maintaining symptoms e.g. (9, 116). Studies also demonstrate associations between facets of IS and specific OC symptom dimensions, suggesting that interoception might be particularly relevant for certain presentations (34, 81, 82, 108). For example, the positive associations of the symmetry/ordering dimension with self-reported awareness of sensations (34) and negative appraisal of internal sensations (81, 82, 108) could suggest that interoceptive dysfunction is more relevant to this clinical presentation. Given the significant clinical heterogeneity within OCD, such findings are particularly meaningful as they could lead to better treatment matching and more targeted interventions.

## Interoception and Core OCD Phenomena

### Sensory Phenomena

Sensory phenomena (SP) are uncomfortable or aversive sensations that motivate repetitive behaviors. As opposed to compulsions driven by an effort to reduce anxiety or avoid harm, individuals with SP report engaging in repetitive behaviors aimed at reducing discomfort elicited by an inner feeling of incompleteness (INC) or “not just right” experience (NJRE) (117). Sensory phenomena are most commonly assessed via self-report [e.g., Obsessive-Compulsive Trait Core Domains Questionnaire; Not Just Right Experiences Questionnaire-Revised; Symmetry/NJRE subscale on the Dimensional Obsessive-Compulsive Scale (118–120)], or clinical interview [University of São Paulo Sensory Phenomena Scale (121)]. As many as 60–70% of individuals diagnosed with OCD experience some form of sensory symptom in the absence of a specific feared outcome (12, 13, 117, 121–123). Further, studies in both clinical and non-clinical samples have found sensory phenomena to be uniquely associated with OC severity even after controlling for harm avoidance and OC-specific beliefs (122, 124–126). Research suggests sensory phenomena are associated with a number of specific and important clinical characteristics, for example, symmetry, ordering, and arranging symptoms (12, 127, 128). Our group identified a relationship between symmetry/ordering/NJRE symptoms and the tendency to notice and be aware of internal sensation as measured by the MAIA, suggesting that increased IS may contribute to these types of symptoms in OCD (34). There is some evidence to suggest that sensory phenomena may also relate to onset and course of OC symptoms (129). For example, one study found that individuals diagnosed with OCD retrospectively perceived increases in NJRE-related urges as one of the top two clinical characteristics (after stress) that played a role in the transition from sub-threshold symptoms to clinical OCD suggesting the potential role of interoceptive processes in the etiology of the disorder (130).

Findings from two neuroimaging studies in individuals with OCD suggest a relationship between SP and neural regions associated with interoception including the insula, sensorimotor, and somatosensory regions (14, 15). Higher SP severity was associated with greater activity of the mid-posterior insula, as well as somatosensory cortex, orbitofrontal cortex, and lateral prefrontal cortex when individuals with OCD viewed “body-focused” videos (15). Interestingly, greater gray matter volumes in sensorimotor regions were also observed in patients with OCD who reported experiencing SP compared to those who did not (14).

### Premonitory Urge and “Urges-for-Action”

Premonitory urges (PU) are uncomfortable or aversive sensations preceding movements or vocalizations in individuals with tic disorders. Often described as a building up of inner tension or an “itching” or “tingling” in the area of the body that has the tic, PUs are most commonly measured using the self-report Premonitory Urge for Tics Scale [PUTS; (131)]. In TS, PU are related to specific OC symptoms including the symmetry and aggression dimensions (132) and several studies have highlighted the similarity between premonitory urges in TS and sensory phenomena preceding compulsive behavior in OCD (121, 133). Indeed, Brandt et al. (134) found a temporal relationship between premonitory urges and compulsions in patients with OCD, characterized by increasing urge intensity until execution of compulsion, followed by immediate, temporary urge decrease (134).

Limited research demonstrates that interoceptive processes may relate to premonitory urges in adults with Tourette Syndrome. Rae et al. (135) found that interoceptive sensibility (as measured by the BPQ) predicted PU severity. Interoceptive accuracy has been examined in relation to PU in two studies: Ganos et al. (136) reported that IAcc predicted PU severity, whereas Rae et al. (135) did not find a significant correlation. The relatively small sample sizes in both of these investigations (*n* = 19–21) combined with slight differences in task design (e.g., length and timing of individual trials) could have contributed to inconsistent findings.

It has been suggested that urges preceding repetitive behaviors in OCD and TD may be phenomenologically similar to “urges-for-action,” which are everyday sensations that motivate behaviors such as blinking or scratching (137, 138). A core feature of “urges-for-action” is the need to suppress or delay a behavior which builds up over time the longer the behavior is suppressed (139), differentiating these pre-movement experiences from those associated with more intentional and goal-directed behaviors (138). Prior work has indicated that every-day “urges-for-action” activate a network of brain regions including the insula and sensorimotor cortical regions (137, 138, 140–142). Using eyeblink suppression as a model to investigate sensory-based urges in OCD, we observed greater eyeblink suppression failures in patients with OCD compared to controls when asked to suppress eye blinking for a period of 60 s (46). OCD patients showed greater neural activity during blink suppression in a network of regions including the anterior insula, cingulate, striatum, superior/inferior parietal cortex, precuneus, and the lateral occipital cortex (46). Interestingly, many of these brain regions overlapped with those found in studies of everyday “urges-for-action” (138).

### Disgust Proneness

Disgust is a basic emotion that functions to motivate avoidance of potentially harmful stimuli that could cause disease (143, 144). It is associated with a visceral response and physiological signs involving interoceptive processes such as nausea (145). Indeed, interoceptive functioning may contribute to disgust proneness, or the extent to which one not only experiences disgust but also finds it to be aversive (146). Disgust proneness can be further divided into two specific dimensions: Disgust Propensity, the frequency of feeling disgusted, and Disgust Sensitivity, how negatively these experiences are appraised (146). Two common assessments of disgust proneness include The Disgust Propensity and Sensitivity Scale-Revised (147) and The Disgust Scale-Revised (148).

Not surprisingly, in both non-clinical and OCD samples, evidence reliably demonstrates a connection involving disgust-proneness with contamination symptoms and behavioral avoidance (149–157). Further, studies have shown disgust proneness to mediate the relation between OC symptoms and behavioral avoidance (158, 159). The construct of contamination includes both physical and mental contamination. While physical contamination involves the presence of a contact contaminant, mental contamination refers to the internal sensation of “dirtiness” in absence of a contact contamination (e.g., dirt, germs) (157, 160, 161). Mental and contact contamination are closely related, yet diverge not only in terms of antecedents but also differ in regards to the efficacy of washing in relieving these feelings (with washing theorized to alleviate physical contamination more than mental) (156, 157, 161). Although disgust proneness has not been investigated in relation to interoceptive accuracy or sensibility, functional neuroimaging studies identify an association between insula activation and disgust (162, 163).

Compared to controls, individuals with OCD showed greater activity in left and right insula when viewing disgust-inducing images, but did not show different patterns of neural activity when viewing fear/threat-inducing images (164–166). As disgust proneness may also be related to negative affect (155), further neuroimaging studies are required to clarify the neural correlates of the association between disgust and OCD symptoms with measures of affect included as covariates in the model.

## Interoception and OCD Treatment

To date, self-report and neuroimaging investigations provide the most compelling evidence for interoceptive differences in patients with OCD. Further, interoception may be more relevant to specific clinical presentations, including individuals with symptoms of symmetry/ordering motivated by sensory phenomena or contamination/washing driven by visceral feelings of disgust. Beyond self-report data, neuroimaging investigations demonstrate the involvement of key interoceptive regions like the insula in the pathophysiology of sensory phenomena, urges-for-action, and disgust. Given this, looking at interoception and related core OCD phenomena in the context of treatment could provide valuable insights necessary for improving therapeutic outcomes.

### Psychotherapy

Cognitive behavioral therapy (CBT) with exposure and response prevention (ExRP) is the gold standard treatment intervention for OCD (167). Response prevention is the elimination of compulsive/avoidance behaviors and exposure entails repeated, systematic confrontation with distress-inducing stimuli. ExRP is theorized to work through various mechanisms such as habituation (i.e., distress decreases naturally during and between exposure sessions) and expectancy violation [i.e., by approaching a feared situation, one learns that it can be tolerated and rarely leads to a feared outcome (168–170)]. Interestingly, studies investigating ExRP treatment response in individuals with OCD have observed associations between insula activations and treatment response, suggesting that interoceptive mechanisms subserved by the insula may have roles in the therapeutic process of ExRP. A recent investigation using whole-brain network-based statistics in unmedicated individuals with OCD found network alterations involving the anterior insula significantly predicted response to exposure therapy (171). Norman et al. (172) found a trend association between greater baseline anterior insula BOLD activity during cognitive control and better ExRP treatment response. Separately, Nakao et al. found that individuals with OCD who showed improvement following either 12 weeks of fluvoxamine or exposure therapy showed increased BOLD activity in the bilateral insula during a Stroop task and reduced activity in the left posterior insula during symptom provocation compared to baseline neural activity (173). Consistently, reduced BOLD activity in regions including the bilateral insula were also observed during individualized symptom-provocation OCD following ExRP treatment (174).

Although one cannot infer a psychological process from neural data alone (175), such findings do suggest that insula function may impact the efficacy of traditional exposure exercises in OCD even though they tend to focus on situations (*in vivo*) or mental stimuli (imaginal) that elicit fear, rather than target sensory-based symptoms. Still, there is evidence suggesting that patients experiencing sensory phenomena derive greater clinical benefit from ExRP when it is optimized to specifically target those symptoms (176). A recent meta-analysis found that though incompleteness improves moderately during CBT, only a minority (18%) of studies tailored treatment to address sensory-related symptoms. Importantly, moderator analyses showed that when treatment was modified to target incompleteness, there was a greater reduction in incompleteness scores (176). Further, some laboratory and outcome research suggests that learned disgust responses are more resistant to extinction and slower to habituate than fear (177–183). Therefore, individuals with OCD with predominant sensory phenomena or disgust may benefit from therapeutic processes that aim to reduce or ameliorate aspects of interoception. Recently, there has been increasing interest in using exposures to specifically target internal sensations (interoceptive exposure) in OCD (103, 170). In one investigation of transdiagnostic CBT, patients with OCD demonstrated the greatest decreases in physical anxiety sensitivity following the introduction of interoceptive exposures (184). Though more published studies are needed, these findings provide preliminary data to suggest that interoceptive exposures may reduce negative appraisal of physical sensations in OCD (103, 184). Khalsa et al. suggested that creating an “interoceptive profile” of patients through assessment of several organ systems (e.g., cardiovascular, gastrointestinal, etc.) could assist clinicians with personalizing and calibrating “dose” of exposures (59). OCD clinicians must also be familiar with the nature of interoceptive-related features such as sensory phenomena, disgust, anxiety sensitivity, and how they differ from fear in treatment. Interestingly, clinician surveys indicate that only a minority report utilizing interoceptive exposure, suggesting that treatment delivery may be suboptimal for many patients with OCD (185, 186).

### Pharmacotherapy

Serotonin reuptake inhibitors (SRIs) including the tricyclic antidepressant clomipramine, and selective SRIs are considered a first line treatment for OCD (167, 187). However, many patients don't respond to an adequate trial and relapse is common after discontinuation (167). Therefore, examining moderators of response to these medications is necessary. Unfortunately, very little has been published on interoception in OCD and treatment response to SRIs. One open trial found that patients reporting sensory phenomena responded better to clomipramine than patients without sensory phenomena (188). Separately, Nakao et al. found that individuals with OCD who showed improvement following 12 weeks of fluvoxamine pharmacotherapy showed increased BOLD activity in the bilateral insula during a cognitive inhibition task (Stroop) and reduced activity in the left posterior insula during symptom provocation compared to baseline neural activity (173). Given the scarcity of research, looking to novel pharmacological treatments that specifically target interoceptive dysfunction may hold promise. Ondansetron, a 5-HT3 antagonist that is FDA-approved for the treatment of nausea and vomiting, demonstrates efficacy in the treatment of sensory symptoms related to pruritus (189). We have found that single high doses of ondansetron reduce activation in the insula, sensorimotor regions, and cingulate cortex in healthy individuals (190). Dopaminergic agents may also hold promise for modulating interoception. Domperidone, a D2 receptor antagonist, was recently found to influence oculomotor avoidance of disgusting visual stimuli (191). Given the research suggesting disgust may be more resistant to habituation than fear, domperidone could may hold potential for augmenting ExRP (191). Botulinum toxin is a protein that acts to block presynaptic release of the neurotransmitter acetylcholine from motor neurons. Though not yet investigated in OCD, it has been shown to reduce premonitory urge and premonitory sensations (generalized urges, tingling sensations) in Tourette's Syndrome (192). To our knowledge, no work-to-date has investigated glutmatate-modulating-agents such as N-acetylcysteine (NAC) on interoception in OCD. However, a prior study reporting no significant effect of NAC on overall OCD symptom severity proposed that this agent might be particular efficacious for patients with urges and sensory phenomena (193) based on prior work revealing NAC efficacy in reducing urges in trichotillomania and excoriation disorder (194, 195).

### Brain Stimulation/Neuromodulation

Deep brain stimulation (DBS) involves the electrical stimulation of specific brain areas through implantation of electrodes. Most commonly used in the treatment of movement disorders such as Parkinson's disease, DBS is a FDA-approved intervention for treatment-refractory OCD (196, 197). Electrodes are most commonly implanted in striatal areas or the subthalamic nucleus (STN) and OCD symptom improvement has been associated with normalization of frontostriatal activity (197–199). Although no studies have directly examined the impact of DBS on interoceptive processing in OCD, neuroimaging findings demonstrate effects of DBS on the functioning of key interoceptive regions. Indeed, resting state functional connectivity with insular and sensorimotor regions at baseline has shown to predict optimal DBS outcome, regardless of target placement (200). Further, DBS of the ventral anterior limb of the internal capsule in patients with treatment-refractory OCD has been found to lead to decreased latero-basal amygdala-insula connectivity (201). Despite these neuroimaging findings, there is a paucity of data investigating the impact of DBS on interoception.

Transcranial magnetic stimulation (TMS) is a non-invasive neuromodulation technique that involves placing a magnetic coil on the scalp that generates a brief and high-intensity magnetic field that excites or inhibits a part of the brain under the coil (202, 203). TMS is FDA-approved for therapeutic applications in several psychiatric conditions such as depression and OCD, although its therapeutic effects on symptoms relating to interoception remain relatively under-investigated. Prior studies have applied inhibitory TMS targeting neural regions known to be involved in interoception, including the anterior insula, somatosensory cortex, and supplementary motor area. In a sample of healthy individuals, inhibitory stimulation using a figure-of-eight coil applied separately over the right anterior insula and right somatosensory area led to reduced interoceptive accuracy and increased interoceptive sensibility (100). These results must be interpreted with caution, as the insula is located approximately 5 cm under the skull, and the standard stimulation protocol used by the authors may not have reached the depth of insula (204). Direct stimulation of the insula may be achieved using different coil configurations, such as H-coils, that can deliver deeper but broader stimulation to the brain (205–207). Existing studies that applied H-coils targeting the insula for addiction (208), severe and enduring anorexia nervosa (209), and eyeblink suppression (210) have reported mixed therapeutic effects of insula stimulation. A recent transcranial direct current stimulation (tDCS) study found that sham, but not anodal stimulation targeting the insula, was related to IAcc improvement (211). Further studies are required to evaluate the clinical efficacy of tDCS and deep TMS.

Although studies targeting the insula with TMS are somewhat difficult to conduct, a body of research has pointed to the potential utility of targeting sensorimotor areas closer to the surface of the brain such as the supplementary motor area (SMA). An investigation applying inhibitory repetitive TMS over the bilateral SMA area for 10 daily sessions in a small sample of individuals with treatment-resistant OCD (*n* = 5) or Tourette syndrome (*n* = 3) (212) found that patients with OCD showed symptom reduction, and two out of three patients with Tourette syndrome showed complete remission of tics at the end of 2 weeks. Significant reductions in anxiety and depressive symptoms were also observed in this study. Subsequent investigations involving the supplementary motor area (SMA) also reported reduction in OCD severity (213–215), with benefits persisting at 6–12 weeks after treatment (214). A recent investigation, also involving inhibitory repetitive TMS of the SMA in individuals with OCD, showed symptom reduction that persists up to 3 months post TMS (216). In this study, both baseline and post-TMS symptom scores predicted post-TMS reduction in functional connectivity of the supplementary motor area with regions including the orbitofrontal cortex, anterior cingulate, and insula (216).

Even though the bulk of existing studies using TMS in OCD did not specifically evaluate changes in interoceptive processes, existing findings indicate that regions that are important in interoception could be indirectly (in the case of insula) or directly (in the case of sensorimotor regions) modulated by TMS (216). A recent randomized-controlled investigation using tDCS found that anodal stimulation of the SMA resulted in superior reductions in OCD symptoms compared to sham in a treatment-resistant sample (217). Despite preliminary evidence that non-invasive neurostimulation techniques like tDCS and transcutaneous electrical nerve stimulation (TENS) may provide therapeutic benefits (218), their application to modulate interoceptive processes is currently lacking.

### Biofeedback and Real-Time fMRI Neurofeedback

Biofeedback generally involves measuring one's own physiological state and feeding the information back in real-time via visual or auditory or tactile feedback so that the individual can learn to modulate the physiological processes that are usually otherwise involuntary (219, 220). Biofeedback has shown promise in ameliorating stress and anxiety symptoms (221–224), and studies also reported improvements in interoceptive accuracy following biofeedback training (225, 226). For example, Meyerholz et al. (225) examined the effect of true cardiac feedback, false-feedback, mindfulness practice, or a waiting control condition on cardiac IAcc. IAcc only improved significantly in the feedback condition, and this change was significantly greater than the three other conditions, suggesting that biofeedback holds promise for modifying interoceptive accuracy (225).

Real-time fMRI neurofeedback, another personalized approach, involves analyzing BOLD activity in real time as fMRI data is collected, and presenting information about neural activity in specific regions to the individual to guide modulation or self-regulation (227). Neurofeedback studies have shown that training can be effective for both modulating anterior insula activity (228–231). In a sample of 3 individuals with OCD with contamination-related obsessions and compulsions, Buyuturkoglu et al. (232) showed that active down-regulation of the insula led to reduced disgust levels and anxiety in response to viewing disgust-inducing images in 2 out of 3 patients. Although further research is required in larger samples, early evidence indicates that real-time fMRI neurofeedback may be beneficial in modulating interoceptive processes by actively regulating neural activity.

## Conclusion

Despite increased understanding in the pathophysiology of OCD, current mainstay treatments have largely remained unchanged over the past 30 years. The clinical heterogeneity of a significant number of patients has not been fully accounted for by traditional anxiety-based models, thus prompting more research into the processing of internal sensations. Interoception presents itself as a promising target for OCD research given the established theoretical framework and measurable behavioral and biological correlates (59). Indeed, a growing body of behavioral and neurobiological literature provides evidence for the role of interoception in OCD. We expect that continuing this line of research will prove useful for both improving personalization of existing treatments like ExRP and identifying new targets for intervention.

## Author Contributions

LB, ES, and GE contributed to the conception of the review. LB and AB searched and reviewed relevant literature. LB wrote the first draft of the manuscript. GE, ES, and KC wrote sections of the manuscript. All authors contributed to manuscript revision, read, and approved the submitted version.

## Conflict of Interest

The authors declare that the research was conducted in the absence of any commercial or financial relationships that could be construed as a potential conflict of interest.

## Publisher's Note

All claims expressed in this article are solely those of the authors and do not necessarily represent those of their affiliated organizations, or those of the publisher, the editors and the reviewers. Any product that may be evaluated in this article, or claim that may be made by its manufacturer, is not guaranteed or endorsed by the publisher.

## References

[1] Kessler RC Petukhova M Sampson NA Zaslavsky AM Wittchen HU. Twelve-month and lifetime prevalence and lifetime morbid risk of anxiety and mood disorders in the United States. Int J Methods Psychiatr Res. (2012) 21:169–84. 10.1002/mpr.1359 22865617 PMC4005415

[2] Eisen JL Sibrava NJ Boisseau CL Mancebo MC Stout RL Pinto A. Five-year course of obsessive-compulsive disorder: predictors of remission and relapse. J Clin Psychiatry. (2013) 74:233–9. 10.4088/JCP.12m07657 23561228 PMC3899346

[3] DuPont RL Rice DP Miller LS Shiraki SS Rowland CR Harwood HJ. Economic costs of anxiety disorders. Anxiety. (1996) 2:167–72. 10.1002/(SICI)1522-7154(1996)2:4<167::AID-ANXI2>3.0.CO;2-L 9160618

[4] Calamari JE Wiegartz PS Riemann BC Cohen RJ Greer A Jacobi DM. Obsessive-compulsive disorder subtypes: an attempted replication and extension of a symptom-based taxonomy. Behav Res Ther. (2004) 42:647–70. 10.1016/S0005-7967(03)00173-6 15081882

[5] Summerfeldt LJ Richter MA Antony MM Swinson RP. Symptom structure in obsessive-compulsive disorder: a confirmatory factor-analytic study. Behav Res Ther. (1999) 37:297–311. 10.1016/S0005-7967(98)00134-X 10204276

[6] Abramowitz JS. The psychological treatment of obsessive-compulsive disorder. Can J Psychiatry. (2006) 51:407–16. 10.1177/070674370605100702 16838822

[7] De Haan E. Effective treatment of OCD? J Am Acad Child Adolesc Psychiatry. (2006) 45:383; author reply 383-4. 10.1097/01.chi.0000205697.73873.c1 16601639

[8] Rachman S Rachman SJ Hodgson RJ. Obsessions and Compulsions. Englewood Cliffs, NJ: Prentice Hall (1980).

[9] Rachman S. A Cognitive Theory of Obsessions. Behavior and Cognitive Therapy Today. Oxford: Elsevier; (1998) p. 209–22.

[10] Salkovskis P Shafran R Rachman S Freeston MH. Multiple pathways to inflated responsibility beliefs in obsessional problems: possible origins and implications for therapy and research. Behav Res Ther. (1999) 37:1055–72. 10.1016/S0005-7967(99)00063-7 10500320

[11] Frost RO Steketee G. Cognitive Approaches to Obsessions and Compulsions: Theory, Assessment, and Treatment. 1st ed. Amsterdam; New York, NY: Pergamon (2002). p. 516.

[12] Ferrao YA Shavitt RG Prado H Fontenelle LF Malavazzi DM de Mathis MA. Sensory phenomena associated with repetitive behaviors in obsessive-compulsive disorder: an exploratory study of 1001 patients. Psychiatry Res. (2012) 197:253–8. 10.1016/j.psychres.2011.09.017 22361443

[13] Summerfeldt LJ. Treating Incompleteness, Ordering, and Arranging Concerns. Washington, DC: American Psychological Association (2007).

[14] Subira M Sato JR Alonso P do Rosario MC Segalas C Batistuzzo MC. Brain structural correlates of sensory phenomena in patients with obsessive-compulsive disorder. J Psychiatry Neurosci. (2015) 40:232–40. 10.1503/jpn.140118 25652753 PMC4478056

[15] Brown C Shahab R Collins K Fleysher L Goodman WK Burdick KE. Functional neural mechanisms of sensory phenomena in obsessive-compulsive disorder. J Psychiatr Res. (2019) 109:68–75. 10.1016/j.jpsychires.2018.11.018 30508745 PMC6347462

[16] Grimaldi SJ Stern ER. Sensory processing and intolerance in OCD. Obsessive-Compulsive Disorder. (2017) 2017:113–8. 10.1093/med/9780190228163.003.0011

[17] Collins K Grimaldi S Stern ER. Sensory Intolerance in OCD. In: McKay D. Storch E. A. Abramowitz J, editors. *Complexities in Obsessive Compulsive and Related Disorders: Advances in Conceptualisation & Treatment*. Oxford: Oxford University Press (2021).

[18] Khalsa SS Adolphs R Cameron OG Critchley HD Davenport PW Feinstein JS. Interoception and mental health: a roadmap. Biol Psychiatry Cogn Neurosci Neuroimaging. (2018) 3:501–13. 10.1016/j.bpsc.2018.04.007 29884281 PMC6054486

[19] Tsakiris M Critchley H. Interoception beyond homeostasis: affect, cognition and mental health. Philos Trans R Soc Lond B Biol Sci. (2016) 371:20160002. 10.1098/rstb.2016.0002 28080961 PMC5062092

[20] Craig AD. How do you feel? interoception: the sense of the physiological condition of the body. Nat Rev Neurosci. (2002) 3:655–66. 10.1038/nrn894 12154366

[21] Critchley HD Harrison NA. Visceral influences on brain and behavior. Neuron. (2013) 77:624–38. 10.1016/j.neuron.2013.02.008 23439117

[22] Paulus MP Stein MB. Interoception in anxiety and depression. Brain Struct Funct. (2010) 214:451–63. 10.1007/s00429-010-0258-9 20490545 PMC2886901

[23] Singer T Critchley HD Preuschoff K. A common role of insula in feelings, empathy and uncertainty. Trends Cogn Sci. (2009) 13:334–40. 10.1016/j.tics.2009.05.001 19643659

[24] Darwin C. Origin of certain instincts. Nature. (1873) 7:417–8. 10.1038/007417a0

[25] James W. What is an emotion? Mind. (1884) 9:188–205. 10.1093/mind/os-IX.34.188

[26] Schachter S Singer JE. Cognitive, social, and physiological determinants of emotional state. Psychol Rev. (1962) 69:379–99. 10.1037/h0046234 14497895

[27] Damasio A. Descartes Error: Emotion, Reason, and the Human Brain. New York, NY: G.P. Putnam (1994).

[28] Ardizzi M Ambrosecchia M Buratta L Ferri F Peciccia M Donnari S. Interoception and positive symptoms in schizophrenia. Front Hum Neurosci. (2016) 10:379. 10.3389/fnhum.2016.00379 27512369 PMC4961721

[29] Brown TA Berner LA Jones MD Reilly EE Cusack A Anderson LK. Psychometric evaluation and norms for the Multidimensional Assessment of Interoceptive Awareness (MAIA) in a clinical eating disorders sample. Eur Eat Disord Rev. (2017) 25:411–6. 10.1002/erv.2532 28714581

[30] Stern ER. Neural circuitry of interoception: new insights into anxiety and obsessive-compulsive disorders. Curr Treat Options Psychiatry. (2014) 1:235–47. 10.1007/s40501-014-0019-0 33344105 PMC7747958

[31] Demartini B Nisticò V Ranieri R Scattolini C Fior G Priori A. Reduced interoceptive accuracy in patients with obsessive–compulsive disorder: A case-control study. J Clin Neurosci. (2021) 90:152–4. 10.1016/j.jocn.2021.05.067 34275541

[32] Schultchen D Zaudig M Krauseneck T Berberich G Pollatos O. Interoceptive deficits in patients with obsessive-compulsive disorder in the time course of cognitive-behavioral therapy. PLoS ONE. (2019) 14:e0217237. 10.1371/journal.pone.0217237 31125377 PMC6534313

[33] Yoris A Esteves S Couto B Melloni M Kichic R Cetkovich M. The roles of interoceptive sensitivity and metacognitive interoception in panic. Behav Brain Funct. (2015) 11:14. 10.1186/s12993-015-0058-8 25889157 PMC4422149

[34] Eng GK Collins KA Brown C Ludlow M Tobe RH Iosifescu DV. Dimensions of interoception in obsessive-compulsive disorder. J Obsessive Compuls Relat Disord. (2020) 27:100584. 10.1016/j.jocrd.2020.100584 33194538 PMC7665060

[35] Lazarov A Liberman N Hermesh H Dar R. Seeking proxies for internal states in obsessive-compulsive disorder. J Abnorm Psychol. (2014) 123:695–704. 10.1037/abn0000004 25133987

[36] Light A Perl E. Reexamination of the dorsal root projection to the spinal dorsal horn including observations on the differential termination of coarse and fine fibers. J Comp Neurol. (1979) 186:117–31. 10.1002/cne.901860202 447880

[37] Saper CB Lowell BB. The hypothalamus. Curr Biol. (2014) 24:R1111–6. 10.1016/j.cub.2014.10.023 25465326

[38] Cameron OG. Interoception: the inside story–a model for psychosomatic processes. Psychosom Med. (2001) 63:697–710. 10.1097/00006842-200109000-00001 11573016

[39] Craig AD. Interoception: the sense of the physiological condition of the body. Curr Opin Neurobiol. (2003) 13:500–5. 10.1016/S0959-4388(03)00090-4 12965300

[40] Craig AD. How do you feel–now? the anterior insula and human awareness. Nat Rev Neurosci. (2009) 10:59–70. 10.1038/nrn2555 19096369

[41] Critchley HD Wiens S Rotshtein P Ohman A Dolan RJ. Neural systems supporting interoceptive awareness. Nat Neurosci. (2004) 7:189–95. 10.1038/nn1176 14730305

[42] Jabbi M Bastiaansen J Keysers C. A common anterior insula representation of disgust observation, experience and imagination shows divergent functional connectivity pathways. PLoS ONE. (2008) 3:e2939. 10.1371/journal.pone.0002939 18698355 PMC2491556

[43] Tanabe J Regner M Sakai J Martinez D Gowin J. Neuroimaging reward, craving, learning, and cognitive control in substance use disorders: review and implications for treatment. Br J Radiol. (2019) 92:20180942. 10.1259/bjr.20180942 30855982 PMC6732921

[44] Naqvi NH Bechara A. The insula and drug addiction: an interoceptive view of pleasure, urges, and decision-making. Brain Struct Funct. (2010) 214:435–50. 10.1007/s00429-010-0268-7 20512364 PMC3698865

[45] Brooks JC Nurmikko TJ Bimson WE Singh KD Roberts N. fMRI of thermal pain: effects of stimulus laterality and attention. Neuroimage. (2002) 15:293–301. 10.1006/nimg.2001.0974 11798266

[46] Stern ER Brown C Ludlow M Shahab R Collins K Lieval A. The buildup of an urge in obsessive-compulsive disorder: behavioral and neuroimaging correlates. Hum Brain Mapp. (2020) 41:1611–25. 10.1002/hbm.24898 31916668 PMC7082184

[47] Tinaz S Malone P Hallett M Horovitz SG. Role of the right dorsal anterior insula in the urge to tic in Tourette syndrome. Mov Disord. (2015) 30:1190–7. 10.1002/mds.26230 25855089 PMC5088605

[48] Deen B Pitskel NB Pelphrey KA. Three systems of insular functional connectivity identified with cluster analysis. Cereb Cortex. (2011) 21:1498–506. 10.1093/cercor/bhq186 21097516 PMC3116731

[49] Cauda F D'Agata F Sacco K Duca S Geminiani G Vercelli A. Functional connectivity of the insula in the resting brain. NeuroImage. (2011) 55:8–23. 10.1016/j.neuroimage.2010.11.049 21111053

[50] Touroutoglou A Hollenbeck M Dickerson BC Barrett LF. Dissociable large-scale networks anchored in the right anterior insula subserve affective experience and attention. Neuroimage. (2012) 60:1947–58. 10.1016/j.neuroimage.2012.02.012 22361166 PMC3345941

[51] Chang LJ Yarkoni T Khaw MW Sanfey AG. Decoding the role of the insula in human cognition: functional parcellation and large-scale reverse inference. Cereb Cortex. (2013) 23:739–49. 10.1093/cercor/bhs065 22437053 PMC3563343

[52] Mutschler I Wieckhorst B Kowalevski S Derix J Wentlandt J Schulze-Bonhage A. Functional organization of the human anterior insular cortex. Neurosci Lett. (2009) 457:66–70. 10.1016/j.neulet.2009.03.101 19429164

[53] Menon V Uddin LQ. Saliency, switching, attention and control: a network model of insula function. Brain Struct Funct. (2010) 214:655–67. 10.1007/s00429-010-0262-0 20512370 PMC2899886

[54] Seeley WW Menon V Schatzberg AF Keller J Glover GH Kenna H. Dissociable intrinsic connectivity networks for salience processing and executive control. J Neurosci. (2007) 27:2349–56. 10.1523/JNEUROSCI.5587-06.2007 17329432 PMC2680293

[55] Sridharan D Levitin DJ Menon V. A critical role for the right fronto-insular cortex in switching between central-executive and default-mode networks. Proc Natl Acad Sci USA. (2008) 105:12569–74. 10.1073/pnas.0800005105 18723676 PMC2527952

[56] Taylor KS Seminowicz DA Davis KD. Two systems of resting state connectivity between the insula and cingulate cortex. Hum Brain Mapp. (2009) 30:2731–45. 10.1002/hbm.20705 19072897 PMC6871122

[57] Namkung H Kim S-H Sawa A. The insula: an underestimated brain area in clinical neuroscience, psychiatry, and neurology. Trends Neurosci. (2017) 40:200–7. 10.1016/j.tins.2017.02.002 28314446 PMC5538352

[58] Berntson GG Khalsa SS. Neural circuits of interoception. Trends Neurosci. (2021) 44:17–28. 10.1016/j.tins.2020.09.011 33378653 PMC8054704

[59] Khalsa SS Lapidus RC. Can interoception improve the pragmatic search for biomarkers in psychiatry? Front Psychiatry. (2016) 7:121. 10.3389/fpsyt.2016.00121 27504098 PMC4958623

[60] Simmons WK Avery JA Barcalow JC Bodurka J Drevets WC Bellgowan P. Keeping the body in mind: Insula functional organization and functional connectivity integrate interoceptive, exteroceptive, and emotional awareness. Hum Brain Mapp. (2013) 34:2944–58. 10.1002/hbm.22113 22696421 PMC6870113

[61] Farb NA Segal ZV Anderson AK. Attentional modulation of primary interoceptive and exteroceptive cortices. Cereb Cortex. (2013) 23:114–26. 10.1093/cercor/bhr385 22267308 PMC3513954

[62] Khalsa SS Rudrauf D Damasio AR Davidson RJ Lutz A Tranel D. Interoceptive awareness in experienced meditators. Psychophysiology. (2008) 45:671–7. 10.1111/j.1469-8986.2008.00666.x 18503485 PMC2637372

[63] Wang X Wu Q Egan L Gu X Liu P Gu H. Anterior insular cortex plays a critical role in interoceptive attention. Elife. (2019) 8:e42265. 10.7554/eLife.42265 30985277 PMC6488299

[64] Khalsa SS Craske MG Li W Vangala S Strober M Feusner JD. Altered interoceptive awareness in anorexia nervosa: effects of meal anticipation, consumption and bodily arousal. Int J Eat Disord. (2015) 48:889–97. 10.1002/eat.22387 25712775 PMC4898968

[65] Aziz Q Thompson D Ng V Hamdy S Sarkar S Brammer M. Cortical processing of human somatic and visceral sensation. J Neurosci. (2000) 20:2657–63. 10.1523/JNEUROSCI.20-07-02657.2000 10729346 PMC6772246

[66] Schandry R Bestler M Montoya P. On the relation between cardiodynamics and heartbeat perception. Psychophysiology. (1993) 30:467–74. 10.1111/j.1469-8986.1993.tb02070.x 8416073

[67] Khalsa SS Rudrauf D Feinstein JS Tranel D. The pathways of interoceptive awareness. Nat Neurosci. (2009) 12:1494–6. 10.1038/nn.2411 19881506 PMC2787640

[68] Bechara A Naqvi N. Listening to your heart: interoceptive awareness as a gateway to feeling. Nat Neurosci. (2004) 7:102–3. 10.1038/nn0204-102 14747831

[69] Koeppel CJ Ruser P Kitzler H Hummel T Croy I. Interoceptive accuracy and its impact on neuronal responses to olfactory stimulation in the insular cortex. Hum Brain Mapp. (2020) 41:2898–908. 10.1002/hbm.24985 32216126 PMC7336161

[70] Yoris A Garcia AM Traiber L Santamaria-Garcia H Martorell M Alifano F. The inner world of overactive monitoring: neural markers of interoception in obsessive-compulsive disorder. Psychol Med. (2017) 47:1957–70. 10.1017/S0033291717000368 28374658

[71] Garfinkel SN Seth AK Barrett AB Suzuki K Critchley HD. Knowing your own heart: distinguishing interoceptive accuracy from interoceptive awareness. Biol Psychol. (2015) 104:65–74. 10.1016/j.biopsycho.2014.11.004 25451381

[72] Mehling WE Price C Daubenmier JJ Acree M Bartmess E Stewart A. the Multidimensional Assessment of Interoceptive Awareness (MAIA). PLoS ONE. (2012) 7:e48230. 10.1371/journal.pone.0048230 23133619 PMC3486814

[73] Porges S. Body Perception Questionnaire: Laboratory of Development Assessment. University of Maryland (1993).

[74] Shields SA Mallory ME Simon A. The body awareness questionnaire: reliability and validity. J Pers Assess. (1989) 53:802–15. 10.1207/s15327752jpa5304_1632566989

[75] Stern ER Grimaldi SJ Muratore A Murrough J Leibu E Fleysher L. Neural correlates of interoception: effects of interoceptive focus and relationship to dimensional measures of body awareness. Hum Brain Mapp. (2017) 38:6068–82. 10.1002/hbm.23811 28901713 PMC5757871

[76] Wang X Tan Y Van den Bergh O von Leupoldt A Qiu J. Intrinsic functional brain connectivity patterns underlying enhanced interoceptive sensibility. J Affect Disord. (2020) 276:804–14. 10.1016/j.jad.2020.07.032 32738665

[77] Grossi D Longarzo M Quarantelli M Salvatore E Cavaliere C De Luca P. Altered functional connectivity of interoception in illness anxiety disorder. Cortex. (2017) 86:22–32. 10.1016/j.cortex.2016.10.018 27871020

[78] Longarzo M Quarantelli M Aiello M Romano M Del Prete A Cimminiello C. The influence of interoceptive awareness on functional connectivity in patients with irritable bowel syndrome. Brain Imaging Behav. (2017) 11:1117–28. 10.1007/s11682-016-9595-5 27704405

[79] Reiss S Peterson RA Gursky DM McNally RJ. Anxiety sensitivity, anxiety frequency and the prediction of fearfulness. Behav Res Ther. (1986) 24:1–8. 10.1016/0005-7967(86)90143-9 3947307

[80] Deacon B Abramowitz J. Anxiety sensitivity and its dimensions across the anxiety disorders. J Anxiety Disord. (2006) 20:837–57. 10.1016/j.janxdis.2006.01.003 16466904

[81] Poli A Melli G Ghisi M Bottesi G Sica C. Anxiety sensitivity and obsessive-compulsive symptom dimensions: further evidence of specific relationships in a clinical sample. Pers Individ Dif. (2017) 109:130–6. 10.1016/j.paid.2017.01.002

[82] Raines AM Oglesby ME Capron DW Schmidt NB. Obsessive compulsive disorder and anxiety sensitivity: identification of specific relations among symptom dimensions. J Obsessive Compulsive Relat Disord. (2014) 3:71–6. 10.1016/j.jocrd.2014.01.001

[83] Ferentzi E Drew R Tihanyi BT Köteles F. Interoceptive accuracy and body awareness–Temporal and longitudinal associations in a non-clinical sample. Physiol Behav. (2018) 184:100–7. 10.1016/j.physbeh.2017.11.015 29155249

[84] Cali G Ambrosini E Picconi L Mehling WE Committeri G. Investigating the relationship between interoceptive accuracy, interoceptive awareness, and emotional susceptibility. Front Psychol. (2015) 6:1202. 10.3389/fpsyg.2015.01202 26379571 PMC4547010

[85] Forkmann T Scherer A Meessen J Michal M Schachinger H Vogele C. Making sense of what you sense: disentangling interoceptive awareness, sensibility and accuracy. Int J Psychophysiol. (2016) 109:71–80. 10.1016/j.ijpsycho.2016.09.019 27702644

[86] Zamariola G Vlemincx E Corneille O Luminet O. Relationship between interoceptive accuracy, interoceptive sensibility, and alexithymia. Pers Individ Dif. (2018) 125:14–20. 10.1016/j.paid.2017.12.02431380655

[87] Bornemann B Herbert BM Mehling WE Singer T. Differential changes in self-reported aspects of interoceptive awareness through 3 months of contemplative training. Front Psychol. (2015) 5:1504. 10.3389/fpsyg.2014.01504 25610410 PMC4284997

[88] Cabrera A Kolacz J Pailhez G Bulbena-Cabre A Bulbena A Porges SW. Assessing body awareness and autonomic reactivity: factor structure and psychometric properties of the Body Perception Questionnaire-Short Form (BPQ-SF). Int J Methods Psychiatric Res. (2018) 27:e1596. 10.1002/mpr.1596 29193423 PMC6877116

[89] Taylor S Zvolensky MJ Cox BJ Deacon B Heimberg RG Ledley DR. Robust dimensions of anxiety sensitivity: development and initial validation of the Anxiety Sensitivity Index-3. Psychol Assess. (2007) 19:176. 10.1037/1040-3590.19.2.176 17563199

[90] Farris SG DiBello AM Allan NP Hogan J Schmidt NB Zvolensky MJ. Evaluation of the Anxiety Sensitivity Index-3 among treatment-seeking smokers. Psychol Assess. (2015) 27:1123–8. 10.1037/pas0000112 25894700 PMC4549201

[91] Pollatos O Kirsch W Schandry R. On the relationship between interoceptive awareness, emotional experience, and brain processes. Cogn Brain Res. (2005) 25:948–62. 10.1016/j.cogbrainres.2005.09.019 16298111

[92] Pollatos O Gramann K Schandry R. Neural systems connecting interoceptive awareness and feelings. Hum Brain Mapp. (2007) 28:9–18. 10.1002/hbm.20258 16729289 PMC6871500

[93] Herbert BM Pollatos O Schandry R. Interoceptive sensitivity and emotion processing: an EEG study. Int J Psychophysiol. (2007) 65:214–27. 10.1016/j.ijpsycho.2007.04.007 17543405

[94] Chong JSX Ng GJP Lee SC Zhou J. Salience network connectivity in the insula is associated with individual differences in interoceptive accuracy. Brain Struct Funct. (2017) 222:1635–44. 10.1007/s00429-016-1297-7 27573028

[95] Haruki Y Ogawa K. Role of anatomical insular subdivisions in interoception: interoceptive attention and accuracy have dissociable substrates. Eur J Neurosci. (2021) 53:2669–80. 10.1111/ejn.15157 33621360

[96] Garfinkel SN Critchley HD. Interoception, emotion and brain: new insights link internal physiology to social behaviour. Commentary on: “Anterior insular cortex mediates bodily sensibility and social anxiety” by Terasawa et al. (2012). Soc Cogn Affect Neurosci. (2013) 8:231–4. 10.1093/scan/nss140 23482658 PMC3594730

[97] Mehling W. Differentiating attention styles and regulatory aspects of self-reported interoceptive sensibility. Philos Trans R Soc Lond B Biol Sci. (2016) 371:20160013. 10.1098/rstb.2016.0013 28080970 PMC5062101

[98] Mehling WE Acree M Stewart A Silas J Jones A. The multidimensional assessment of interoceptive awareness, version 2 (MAIA-2). PLoS ONE. (2018) 13:e0208034. 10.1371/journal.pone.0208034 30513087 PMC6279042

[99] Herbert BM Pollatos O. The body in the mind: on the relationship between interoception and embodiment. Top Cogn Sci. (2012) 4:692–704. 10.1111/j.1756-8765.2012.01189.x 22389201

[100] Pollatos O Herbert BM Mai S Kammer T. Changes in interoceptive processes following brain stimulation. Philos Trans R Soc Lond B Biol Sci. (2016) 371:201600016. 10.1098/rstb.2016.0016 28080973 PMC5062104

[101] Jokić B Purić D. Seeking proxies for internal states as a possible alternative for rationality and experientiality. SAGE Open. (2021) 11:2158244020986533. 10.1177/2158244020986533

[102] Peterson RA Reiss S. Anxiety Sensitivity Index manual. 2nd ed. Worthington, OH: International Diagnostic Systems (1992).

[103] Blakey SM Abramowitz JS. Interoceptive exposure: an overlooked modality in the cognitive-behavioral treatment of OCD. Cogn Behav Pract. (2018) 25:145–55. 10.1016/j.cbpra.2017.01.002

[104] Blakey SM Abramowitz JS Reuman L Leonard RC Riemann BC. Anxiety sensitivity as a predictor of outcome in the treatment of obsessive-compulsive disorder. J Behav Ther Exp Psy. (2017) 57:113–7. 10.1016/j.jbtep.2017.05.003 28505489

[105] Naragon-Gainey K. Meta-analysis of the relations of anxiety sensitivity to the depressive and anxiety disorders. Psychol Bull. (2010) 136:128–50. 10.1037/a0018055 20063929

[106] Stanley IH Boffa JW Rogers ML Hom MA Albanese BJ Chu C. Anxiety sensitivity and suicidal ideation/suicide risk: a meta-analysis. J Consult Clin Psychol. (2018) 86:946–60. 10.1037/ccp0000342 30335426 PMC6469498

[107] Vujanovic AA Farris SG Bartlett BA Lyons RC Haller M Colvonen PJ. Anxiety sensitivity in the association between posttraumatic stress and substance use disorders: a systematic review. Clin Psychol Rev. (2018) 62:37–55. 10.1016/j.cpr.2018.05.003 29778929

[108] Calamari JE Rector NA Woodard JL Cohen RJ Chik HM. Anxiety sensitivity and obsessive—compulsive disorder. Assessment. (2008) 15:351–63. 10.1177/1073191107312611 18310595

[109] Krebs G Hannigan L Gregory AM Rijsdijk FV Eley TC. Reciprocal links between anxiety sensitivity and obsessive–compulsive symptoms in youth: a longitudinal twin study. J Child Psychol Psychiatry. (2020) 61:979–87. 10.1111/jcpp.13183 31950513 PMC7497024

[110] Poletti S Radaelli D Cucchi M Ricci L Vai B Smeraldi E. Neural correlates of anxiety sensitivity in panic disorder: a functional magnetic resonance imaging study. Psychiatry Res Neuroimaging. (2015) 233:95–101. 10.1016/j.pscychresns.2015.05.013 26071623

[111] Stein MB Simmons AN Feinstein JS Paulus MP. Increased amygdala and insula activation during emotion processing in anxiety-prone subjects. Am J Psychiat. (2007) 164:318–27. 10.1176/ajp.2007.164.2.318 17267796

[112] Kim M-K Kim B Choi TK Lee S-H. White matter correlates of anxiety sensitivity in panic disorder. J Affect Disord. (2017) 207:148–56. 10.1016/j.jad.2016.08.043 27721189

[113] Paulus MP Stein MB. An insular view of anxiety. Biol Psychiatry. (2006) 60:383–7. 10.1016/j.biopsych.2006.03.042 16780813

[114] Craig AD. Interoception and emotion: A neuroanatomical perspective. In Lewis M Haviland-Jones JM Barrett LF, editors. Handbook of Emotions. New York, NY: The Guilford Press (2008). p. 272–92.

[115] Ceunen E Vlaeyen JW Van Diest I. On the origin of interoception. Front psychol. (2016) 7:743. 10.3389/fpsyg.2016.00743 27242642 PMC4876111

[116] Salkovskis PM. Obsessional-compulsive problems: a cognitive-behavioural analysis. Behav Res Ther. (1985) 23:571–83. 10.1016/0005-7967(85)90105-6 4051930

[117] Rasmussen SA Eisen JL. The epidemiology and differential diagnosis of obsessive-compulsive disorder. In: Zwangsstörungen/obsessive-Compulsive Disorders. (1992). p. 1–14. 10.1007/978-3-642-77608-3_17961532

[118] Coles ME Frost RO Heimberg RG Rhéaume J. “Not just right experiences”: perfectionism, obsessive–compulsive features and general psychopathology. Behav Res Ther. (2003) 41:681–700. 10.1016/S0005-7967(02)00044-X 12732376

[119] Summerfeldt LJ Kloosterman PH Antony MM Swinson RP. Examining an obsessive-compulsive core dimensions model: structural validity of harm avoidance and incompleteness. J Obsessive Compulsive Relat Disord. (2014) 3:83–94. 10.1016/j.jocrd.2014.01.003

[120] Abramowitz JS Deacon BJ Olatunji BO Wheaton MG Berman NC Losardo D. Assessment of obsessive-compulsive symptom dimensions: development and evaluation of the Dimensional Obsessive-Compulsive Scale. Psychol Assess. (2010) 22:180–98. 10.1037/a0018260 20230164

[121] Rosario MC Prado HS Borcato S Diniz JB Shavitt RG Hounie AG. Validation of the university of sáo páulo sensory phenomena scale: initial psychometric properties. CNS Spectr. (2009) 14:315–23. 10.1017/S1092852900020319 19668122

[122] Lee JC Prado HS Diniz JB Borcato S da Silva CB Hounie AG. Perfectionism and sensory phenomena: Phenotypic components of obsessive-compulsive disorder. Compr Psychiatry. (2009) 50:431–6. 10.1016/j.comppsych.2008.11.007 19683613

[123] Shavitt RG de Mathis MA Oki F Ferrao YA Fontenelle LF Torres AR. Phenomenology of OCD: lessons from a large multicenter study and implications for ICD-11. J Psychiatr Res. (2014) 57:141–8. 10.1016/j.jpsychires.2014.06.010 25012187 PMC7326117

[124] Ecker W Gonner S. Incompleteness and harm avoidance in OCD symptom dimensions. Behav Res Ther. (2008) 46:895–904. 10.1016/j.brat.2008.04.002 18514616

[125] Pietrefesa AS Coles ME. Moving beyond an exclusive focus on harm avoidance in obsessive compulsive disorder: considering the role of incompleteness. Behav Ther. (2008) 39:224–31. 10.1016/j.beth.2007.08.004 18721636

[126] Taylor S McKay D Crowe KB Abramowitz JS Conelea CA Calamari JE. The sense of incompleteness as a motivator of obsessive-compulsive symptoms: an empirical analysis of concepts and correlates. Behav Ther. (2014) 45:254–62. 10.1016/j.beth.2013.11.004 24491200 PMC3914013

[127] Coles ME Ravid A. Clinical presentation of not-just right experiences (NJREs) in individuals with OCD: characteristics and response to treatment. Behav Res Ther. (2016) 87:182–7. 10.1016/j.brat.2016.09.013 27716490

[128] Bragdon LB Coles ME. Examining heterogeneity of obsessive-compulsive disorder: evidence for subgroups based on motivations. J Anxiety Disord. (2017) 45:64–71. 10.1016/j.janxdis.2016.12.002 27960103

[129] Sica C Caudek C Rocco Chiri L Ghisi M Marchetti I. “Not just right experiences” predict obsessive–compulsive symptoms in non-clinical Italian individuals: a one-year longitudinal study. J Obsessive Compulsive Relat Disord. (2012) 1:159–67. 10.1016/j.jocrd.2012.03.006

[130] Coles ME Hart AS Schofield CA. Initial data characterizing the progression from obsessions and compulsions to full-blown obsessive compulsive disorder. Cogn Ther Res. (2012) 36:685–93. 10.1007/s10608-011-9404-9

[131] Woods DW Piacentini J Himle MB Chang S. Premonitory Urge for Tics Scale (PUTS): initial psychometric results and examination of the premonitory urge phenomenon in youths with Tic disorders. J Dev Behav Pediatr. (2005) 26:397–403. 10.1097/00004703-200512000-00001 16344654

[132] Kano Y Matsuda N Nonaka M Fujio M Kuwabara H Kono T. Sensory phenomena related to tics, obsessive-compulsive symptoms, and global functioning in Tourette syndrome. Compr Psychiatry. (2015) 62:141–6. 10.1016/j.comppsych.2015.07.006 26343478

[133] Miguel EC do Rosario-Campos MC Prado HS do Valle R Rauch SL Coffey BJ. Sensory phenomena in obsessive-compulsive disorder and Tourette's disorder. J Clin Psychiatry. (2000) 61:150–6; quiz 7. 10.4088/JCP.v61n0213 10732667

[134] Brandt VC Hermanns J Beck C Baumer T Zurowski B Munchau A. The temporal relationship between premonitory urges and covert compulsions in patients with obsessive-compulsive disorder. Psychiatry Res. (2018) 262:6–12. 10.1016/j.psychres.2018.01.041 29407570

[135] Rae CL Larsson DE Garfinkel SN Critchley HD. Dimensions of interoception predict premonitory urges and tic severity in Tourette syndrome. Psychiatry Res. (2019) 271:469–75. 10.1016/j.psychres.2018.12.036 30544073

[136] Ganos C Garrido A Navalpotro-Gomez I Ricciardi L Martino D Edwards MJ. Premonitory urge to tic in Tourette's is associated with interoceptive awareness. Mov Disord. (2015) 30:1198–202. 10.1002/mds.26228 25879819

[137] Berman BD Horovitz SG Morel B Hallett M. Neural correlates of blink suppression and the buildup of a natural bodily urge. Neuroimage. (2012) 59:1441–50. 10.1016/j.neuroimage.2011.08.050 21906689 PMC3230735

[138] Jackson SR Parkinson A Kim SY Schuermann M Eickhoff SB. On the functional anatomy of the urge-for-action. Cogn Neurosci. (2011) 2:227–43. 10.1080/17588928.2011.604717 22299020 PMC3259619

[139] Botteron HE Richards CA Nishino T Ueda K Acevedo HK Koller JM. The urge to blink in Tourette syndrome. Cortex. (2019) 120:556–66. 10.1016/j.cortex.2019.07.010 31525588 PMC6825887

[140] Holle H Warne K Seth AK Critchley HD Ward J. Neural basis of contagious itch and why some people are more prone to it. Proc Natl Acad Sci USA. (2012) 109:19816–21. 10.1073/pnas.1216160109 23150550 PMC3511754

[141] Lerner A Bagic A Hanakawa T Boudreau EA Pagan F Mari Z. Involvement of insula and cingulate cortices in control and suppression of natural urges. Cereb Cortex. (2009) 19:218–23. 10.1093/cercor/bhn074 18469316 PMC2638741

[142] Mazzone SB Cole LJ Ando A Egan GF Farrell MJ. Investigation of the neural control of cough and cough suppression in humans using functional brain imaging. J Neurosci. (2011) 31:2948–58. 10.1523/JNEUROSCI.4597-10.2011 21414916 PMC6623770

[143] Harrison NA Gray MA Gianaros PJ Critchley HD. The embodiment of emotional feelings in the brain. J Neurosci. (2010) 30:12878–84. 10.1523/JNEUROSCI.1725-10.2010 20861391 PMC3044882

[144] Rozin P Fallon AE. A perspective on disgust. Psychol Rev. (1987) 94:23–41. 10.1037/0033-295X.94.1.23 3823304

[145] Izard CE. Patterns of Emotions: A New Analysis of Anxiety and Depression New York, NY: Academic Press (2013).

[146] Van Overveld W De Jong P Peters M Cavanagh K Davey G. Disgust propensity and disgust sensitivity: separate constructs that are differentially related to specific fears. Pers Individ Dif. (2006) 41:1241–52. 10.1016/j.paid.2006.04.021

[147] van Overveld M de Jong PJ Peters ML. The disgust propensity and sensitivity scale–revised: its predictive value for avoidance behavior. Pers Individ Dif. (2010) 49:706–11. 10.1016/j.paid.2010.06.008

[148] Olatunji BO Williams NL Tolin DF Abramowitz JS Sawchuk CN Lohr JM. The disgust scale: item analysis, factor structure, and suggestions for refinement. Psychol Assess. (2007) 19:281. 10.1037/1040-3590.19.3.281 17845120

[149] Olatunji BO Williams NL Lohr JM Sawchuk CN. The structure of disgust: domain specificity in relation to contamination ideation and excessive washing. Behav Res Ther. (2005) 43:1069–86. 10.1016/j.brat.2004.08.002 15967177

[150] Cisler JM Olatunji BO Lohr JM. Disgust, fear, and the anxiety disorders: a critical review. Clin Psychol Rev. (2009) 29:34–46. 10.1016/j.cpr.2008.09.007 18977061 PMC2895912

[151] Woody SR Teachman BA. Intersection of disgust and fear: normative and pathological views. Clin Psychol Sci Pract. (2000) 7:291–311. 10.1093/clipsy.7.3.291

[152] Tolin DF Woods CM Abramowitz JS. Disgust sensitivity and obsessive–compulsive symptoms in a non-clinical sample. J Behav Ther Exp Psy. (2006) 37:30–40. 10.1016/j.jbtep.2005.09.003 16226217

[153] Mancini F Gragnani A D'Olimpio F. The connection between disgust and obsessions and compulsions in a non-clinical sample. Pers Individ Dif. (2001) 31:1173–80. 10.1016/S0191-8869(00)00215-4

[154] Olatunji BO Armstrong T Elwood L. Is disgust proneness associated with anxiety and related disorders? a qualitative review and meta-analysis of group comparison and correlational studies. Perspect Psychol Sci. (2017) 12:613–48. 10.1177/1745691616688879 28651058

[155] Olatunji BO Ebesutani C David B Fan Q McGrath PB. Disgust proneness and obsessive–compulsive symptoms in a clinical sample: structural differentiation from negative affect. J Anxiety Disord. (2011) 25:932–8. 10.1016/j.janxdis.2011.05.006 21696916

[156] Melli G Bulli F Carraresi C Tarantino F Gelli S Poli A. The differential relationship between mental contamination and the core dimensions of contact contamination fear. J Anxiety Disord. (2017) 45:9–16. 10.1016/j.janxdis.2016.11.005 27886574

[157] Melli G Bulli F Carraresi C Stopani E. Disgust propensity and contamination-related OCD symptoms: the mediating role of mental contamination. J Obsessive Compulsive Relat Disord. (2014) 3:77–82. 10.1016/j.jocrd.2014.01.002

[158] Olatunji BO McKay D. Disgust and psychiatric illness: have we remembered? Br J Psychiatry. (2007) 190:457–9. 10.1192/bjp.bp.106.032631 17541102

[159] Deacon B Olatunji BO. Specificity of disgust sensitivity in the prediction of behavioral avoidance in contamination fear. Behav Res Ther. (2007) 45:2110–20. 10.1016/j.brat.2007.03.008 17481576

[160] Rachman S. Fear of Contamination: Assessment & Treatment. New York, NY: Oxford University Press Inc (2006). 10.1093/med:psych/9780199296934.001.0001

[161] Rachman S. Fear of contamination. Behav Res Ther. (2004) 42:1227–55. 10.1016/j.brat.2003.10.009 15381436

[162] Wicker B Keysers C Plailly J Royet JP Gallese V Rizzolatti G. Both of us disgusted in my insula: the common neural basis of seeing and feeling disgust. Neuron. (2003) 40:655–64. 10.1016/S0896-6273(03)00679-2 14642287

[163] Wright P He G Shapira NA Goodman WK Liu Y. Disgust and the insula: fMRI responses to pictures of mutilation and contamination. Neuroreport. (2004) 15:2347–51. 10.1097/00001756-200410250-00009 15640753

[164] Lawrence NS An SK Mataix-Cols D Ruths F Speckens A Phillips ML. Neural responses to facial expressions of disgust but not fear are modulated by washing symptoms in OCD. Biol Psychiatry. (2007) 61:1072–80. 10.1016/j.biopsych.2006.06.033 17097073

[165] Schienle A Schafer A Stark R Walter B Vaitl D. Neural responses of OCD patients towards disorder-relevant, generally disgust-inducing and fear-inducing pictures. Int J Psychophysiol. (2005) 57:69–77. 10.1016/j.ijpsycho.2004.12.013 15935263

[166] Shapira NA Liu Y He AG Bradley MM Lessig MC James GA. Brain activation by disgust-inducing pictures in obsessive-compulsive disorder. Biol Psychiatry. (2003) 54:751–6. 10.1016/S0006-3223(03)00003-9 14512216

[167] Koran LM Hanna GL Hollander E Nestadt G Simpson HB American Psychiatric Association. Practice guideline for the treatment of patients with obsessive-compulsive disorder. Am J Psychiatry. (2007) 164:5–53. 17849776

[168] Foa EB Huppert JD Cahill SP. Emotional processing theory: an update. In: Rothbaum BO, editor. Pathological Anxiety: Emotional Processing in Etiology and Treatment. New York, NY: Guilford (2006) p. 3–24.

[169] Foa EB Kozak MJ. Emotional processing of fear: exposure to corrective information. Psychol Bull. (1986) 99:20. 10.1037/0033-2909.99.1.20 2871574

[170] Craske MG Kircanski K Zelikowsky M Mystkowski J Chowdhury N Baker A. Optimizing inhibitory learning during exposure therapy. Behav Res Ther. (2008) 46:5–27. 10.1016/j.brat.2007.10.003 18005936

[171] Shi TC Pagliaccio D Cyr M Simpson HB Marsh R. Network-based functional connectivity predicts response to exposure therapy in unmedicated adults with obsessive–compulsive disorder. Neuropsychopharmacology. (2021) 46:1035–44. 10.1038/s41386-020-00929-9 33446895 PMC8115173

[172] Norman LJ Mannella KA Yang H Angstadt M Abelson JL Himle JA. Treatment-specific associations between brain activation and symptom reduction in OCD following CBT: a randomized fMRI trial. Am J Psychiat. (2021) 178:39–47. 10.1176/appi.ajp.2020.19080886 32854533 PMC8528223

[173] Nakao T Nakagawa A Yoshiura T Nakatani E Nabeyama M Yoshizato C. A functional MRI comparison of patients with obsessive-compulsive disorder and normal controls during a Chinese character Stroop task. Psychiatry Res. (2005) 139:101–14. 10.1016/j.pscychresns.2004.12.004 15970434

[174] Schiepek G Tominschek I Heinzel S Aigner M Dold M Unger A. Discontinuous patterns of brain activation in the psychotherapy process of obsessive-compulsive disorder: converging results from repeated fMRI and daily self-reports. PLoS ONE. (2013) 8:e71863. 10.1371/journal.pone.0071863 23977168 PMC3744482

[175] Poldrack RA. Can cognitive processes be inferred from neuroimaging data? Trends Cogn Sci. (2006) 10:59–63. 10.1016/j.tics.2005.12.004 16406760

[176] Schwartz RA. Treating incompleteness in obsessive-compulsive disorder: a meta-analytic review. J Obsessive Compulsive Relat Disord. (2018) 19:50–60. 10.1016/j.jocrd.2018.08.001

[177] Smits JA Telch MJ Randall PK. An examination of the decline in fear and disgust during exposure-based treatment. Behav Res Ther. (2002) 40:1243–53. 10.1016/S0005-7967(01)00094-8 12384321

[178] McKay D. Treating disgust reactions in contamination-based obsessive-compulsive disorder. J Behav Ther Exp Psychiatry. (2006) 37:53–9. 10.1016/j.jbtep.2005.09.005 16253206

[179] Olatunji BO Wolitzky-Taylor KB Willems J Lohr JM Armstrong T. Differential habituation of fear and disgust during repeated exposure to threat-relevant stimuli in contamination-based OCD: an analogue study. J Anxiety Disord. (2009) 23:118–23. 10.1016/j.janxdis.2008.04.006 18541403

[180] Mason EC Richardson R. Looking beyond fear: the extinction of other emotions implicated in anxiety disorders. J Anxiety Disord. (2010) 24:63–70. 10.1016/j.janxdis.2009.08.007 19747796

[181] Adams Jr TG Willems JL Bridges AJ. Contamination aversion and repeated exposure to disgusting stimuli. Anxiety Stress Coping. (2011) 24:157–65. 10.1080/10615806.2010.506953 20737324

[182] Rozin P Haidt J McCauley CR. Disgust. In: Lewis M Haviland-Jones JM Barrett LF, editors. Handbook of Emotions, 3rd ed. New York, NY: Guilford Press (2008). p. 757–76

[183] Olatunji BO Smits JA Connolly K Willems J Lohr JM. Examination of the decline in fear and disgust during exposure to threat-relevant stimuli in blood–injection–injury phobia. J Anxiety Disord. (2007) 21:445–55. 10.1016/j.janxdis.2006.05.001 16806801

[184] Boswell JF Farchione TJ Sauer-Zavala S Murray HW Fortune MR Barlow DH. Anxiety sensitivity and interoceptive exposure: a transdiagnostic construct and change strategy. Behav Ther. (2013) 44:417–31. 10.1016/j.beth.2013.03.006 23768669 PMC3727659

[185] Sars D van Minnen A. On the use of exposure therapy in the treatment of anxiety disorders: a survey among cognitive behavioural therapists in the Netherlands. BMC Psychol. (2015) 3:1–10. 10.1186/s40359-015-0083-2 26246900 PMC4525733

[186] Hipol LJ Deacon BJ. Dissemination of evidence-based practices for anxiety disorders in Wyoming: a survey of practicing psychotherapists. Behav Modif. (2013) 37:170–88. 10.1177/0145445512458794 23012685

[187] Bandelow B Sher L Bunevicius R Hollander E Kasper S Zohar J. Guidelines for the pharmacological treatment of anxiety disorders, obsessive–compulsive disorder and posttraumatic stress disorder in primary care. Int J Psychiatry Clin Pract. (2012) 16:77–84. 10.3109/13651501.2012.667114 22540422

[188] Shavitt RG Belotto C Curi M Hounie AG Rosario-Campos MC Diniz JB. Clinical features associated with treatment response in obsessive-compulsive disorder. Compr Psychiatry. (2006) 47:276–81. 10.1016/j.comppsych.2005.09.001 16769302

[189] Kyriakides K Hussain SK Hobbs GJ. Management of opioid-induced pruritus: a role for 5-HT3 antagonists? Br J Anaesth. (1999) 82:439–41. 10.1093/bja/82.3.439 10434832

[190] Stern ER Shahab R Grimaldi SJ Leibu E Murrough JW Fleysher L. High dose ondansetron reduces activation of interoceptive and sensorimotor brain regions. Neuropsychopharmacology. (2019) 44:390–8. 10.1038/s41386-018-0174-x 30116006 PMC6300545

[191] Nord CL Dalmaijer ES Armstrong T Baker K Dalgleish T. A causal role for gastric rhythm in human disgust avoidance. Curr Biol. (2021) 31:629–34.e3. 10.1016/j.cub.2020.10.087 33238154 PMC7883304

[192] Kwak C Vuong KD Jankovic J. Premonitory sensory phenomenon in Tourette's syndrome. Mov Disord. (2003) 18:1530–3. 10.1002/mds.10618 14673893

[193] Costa DL Diniz JB Requena G Joaquim MA Pittenger C Bloch MH. Randomized, double-blind, placebo-controlled trial of N-acetylcysteine augmentation for treatment-resistant obsessive-compulsive disorder. J Clin Psychiatry. (2017) 78:e766–73. 10.4088/JCP.16m11101 28617566

[194] Grant JE Odlaug BL Kim SW. N-acetylcysteine, a glutamate modulator, in the treatment of trichotillomania: a double-blind, placebo-controlled study. Arch Gen Psychiatry. (2009) 66:756–63. 10.1001/archgenpsychiatry.2009.60 19581567

[195] Grant JE Chamberlain SR Redden SA Leppink EW Odlaug BL Kim SW. N-acetylcysteine in the treatment of excoriation disorder: a randomized clinical trial. JAMA Psychiatry. (2016) 73:490–6. 10.1001/jamapsychiatry.2016.0060 27007062

[196] Hageman SB van Rooijen G Bergfeld IO Schirmbeck F de Koning P Schuurman PR. Deep brain stimulation versus ablative surgery for treatment-refractory obsessive-compulsive disorder: a meta-analysis. Acta Psychiat Scand. (2021) 143:307–18. 10.1111/acps.13276 33492682

[197] Alonso P Cuadras D Gabriëls L Denys D Goodman W Greenberg BD. Deep brain stimulation for obsessive-compulsive disorder: a meta-analysis of treatment outcome and predictors of response. PLoS ONE. (2015) 10:e0133591. 10.1371/journal.pone.0133591 26208305 PMC4514753

[198] Figee M Luigjes J Smolders R Valencia-Alfonso C-E Van Wingen G De Kwaasteniet B. Deep brain stimulation restores frontostriatal network activity in obsessive-compulsive disorder. Nat Neurosci. (2013) 16:386–7. 10.1038/nn.3344 23434914

[199] Goodman WK Storch EA Sheth SA. Harmonizing the neurobiology and treatment of obsessive-compulsive disorder. Am J Psychiatry. (2021) 178:17–29. 10.1176/appi.ajp.2020.20111601 33384007 PMC8091795

[200] Li N Hollunder B Baldermann JC Kibleur A Treu S Akram H. A unified functional network target for deep brain stimulation in obsessive-compulsive disorder. Biol Psychiatry. (2021) S0006-3223(21)01223-3. 10.1016/j.biopsych.2021.04.006 34134839

[201] Fridgeirsson EA Figee M Luigjes J van den Munckhof P Schuurman PR van Wingen G. Deep brain stimulation modulates directional limbic connectivity in obsessive-compulsive disorder. Brain. (2020) 143:1603–12. 10.1093/brain/awaa100 32352147 PMC7241947

[202] Barker AT Jalinous R Freeston IL. Non-invasive magnetic stimulation of human motor cortex. Lancet. (1985) 325:1106–7. 10.1016/S0140-6736(85)92413-4 2860322

[203] Hallett M. Transcranial magnetic stimulation: a primer. Neuron. (2007) 55:187–99. 10.1016/j.neuron.2007.06.026 17640522

[204] Coll M-P Penton T Hobson H. Important methodological issues regarding the use of transcranial magnetic stimulation to investigate interoceptive processing: a comment on Pollatos et al. (2016). Philos Trans R Soc B Biol Sci. (2017) 372:20160506. 10.1098/rstb.2016.0506 28396481 PMC5394648

[205] Deng Z-D Lisanby SH Peterchev AV. Electric field depth–focality tradeoff in transcranial magnetic stimulation: simulation comparison of 50 coil designs. Brain Stimul. (2013) 6:1–13. 10.1016/j.brs.2012.02.005 22483681 PMC3568257

[206] Zangen A Roth Y Voller B Hallett M. Transcranial magnetic stimulation of deep brain regions: evidence for efficacy of the H-coil. Clin Neurophysiol. (2005) 116:775–9. 10.1016/j.clinph.2004.11.008 15792886

[207] Malik S Jacobs M Cho S-S Boileau I Blumberger D Heilig M. Deep TMS of the insula using the H-coil modulates dopamine release: a crossover [11 C] PHNO-PET pilot trial in healthy humans. Brain Imaging Behav. (2018) 12:1306–17. 10.1007/s11682-017-9800-1 29170944

[208] Dinur-Klein L Dannon P Hadar A Rosenberg O Roth Y Kotler M. Smoking cessation induced by deep repetitive transcranial magnetic stimulation of the prefrontal and insular cortices: a prospective, randomized controlled trial. Biol Psychiatry. (2014) 76:742–9. 10.1016/j.biopsych.2014.05.020 25038985

[209] Knyahnytska YO Blumberger DM Daskalakis ZJ Zomorrodi R Kaplan AS. Insula H-coil deep transcranial magnetic stimulation in severe and enduring anorexia nervosa (SE-AN): a pilot study. Neuropsychiatr Dis Treat. (2019) 15:2247–56. 10.2147/NDT.S207630 31496707 PMC6689531

[210] Spagnolo PA Wang H Srivanitchapoom P Schwandt M Heilig M Hallett M. Lack of target engagement following low-frequency deep transcranial magnetic stimulation of the anterior insula. Neuromodulation. (2019) 22:877–83. 10.1111/ner.12875 30370983 PMC6488455

[211] Sagliano L Magliacano A Parazzini M Fiocchi S Trojano L Grossi D. Modulating interoception by insula stimulation: a double-blinded tDCS study. Neurosci Lett. (2019) 696:108–13. 10.1016/j.neulet.2018.12.022 30572103

[212] Mantovani A Lisanby SH Pieraccini F Ulivelli M Castrogiovanni P Rossi S. Repetitive transcranial magnetic stimulation (rTMS) in the treatment of obsessive–compulsive disorder (OCD) and Tourette's syndrome (TS). Int J Neuropsychopharmacol. (2006) 9:95–100. 10.1017/S1461145705005729 15982444

[213] Mantovani A Shubeck J Gowatsky J Simpson HB Greenberg B. P 247. Incompleteness and harm avoidance in obsessive compulsive disorder: different response to transcranial magnetic stimulation. Clin Neurophysiol. (2013) 124:e182–e3. 10.1016/j.clinph.2013.04.323

[214] Mantovani A Simpson HB Fallon BA Rossi S Lisanby SH. Randomized sham-controlled trial of repetitive transcranial magnetic stimulation in treatment-resistant obsessive–compulsive disorder. Int J Neuropsychopharmacol. (2010) 13:217–27. 10.1017/S1461145709990435 19691873

[215] Gomes PVO Brasil-Neto JP Allam N Rodrigues de Souza E. A randomized, double-blind trial of repetitive transcranial magnetic stimulation in obsessive-compulsive disorder with three-month follow-up. J Neuropsychiatry Clin Neurosci. (2012) 24:437–43. 10.1176/appi.neuropsych.11100242 23224449

[216] Mantovani A Neri F D'Urso G Mencarelli L Tatti E Momi D. Functional connectivity changes and symptoms improvement after personalized, double-daily dosing, repetitive transcranial magnetic stimulation in obsessive-compulsive disorder: a pilot study. J Psychiatric Res. (2020) 136:560–70. 10.1016/j.jpsychires.2020.10.030 33158554

[217] Silva RdMFd Brunoni AR Goerigk S Batistuzzo MC Costa DLdC Diniz JB. Efficacy and safety of transcranial direct current stimulation as an add-on treatment for obsessive-compulsive disorder: a randomized, sham-controlled trial. Neuropsychopharmacology. (2021) 87:S127. 10.1016/j.biopsych.2020.02.342 33452434 PMC8115679

[218] Chen WG Schloesser D Arensdorf AM Simmons JM Cui C Valentino R. The emerging science of interoception: sensing, integrating, interpreting, and regulating signals within the self. Trends Neurosci. (2021) 44:3–16. 10.1016/j.tins.2020.10.007 33378655 PMC7780231

[219] Saha S Dey D Bhattacharyya IM Das A editors. An investigation on biofeedback analysis and psychosomatic applications 2015. In: International Conference on Recent Developments in Control, Automation and Power Engineering (RDCAPE) Noida: IEEE (2015). 10.1109/RDCAPE.2015.7281366

[220] Frank DL Khorshid L Kiffer JF Moravec CS McKee MG. Biofeedback in medicine: who, when, why and how? Ment Health Fam Med. (2010) 7:85–91. 22477926 PMC2939454

[221] Weerdmeester J van Rooij MM Engels RC Granic I. An integrative model for the effectiveness of biofeedback interventions for anxiety regulation. J Med Internet Res. (2020) 22:e14958. 10.2196/14958 32706654 PMC7413290

[222] Goessl VC Curtiss JE Hofmann SG. The effect of heart rate variability biofeedback training on stress and anxiety: a meta-analysis. Psychol Med. (2017) 47:2578–86. 10.1017/S0033291717001003 28478782

[223] Schoenberg PL David AS. Biofeedback for psychiatric disorders: a systematic review. Appl Psychophysiol Biofeedback. (2014) 39:109–35. 10.1007/s10484-014-9246-9 24806535

[224] Yucha C Montgomery D. Evidence-Based Practice in Biofeedback and Neurofeedback. AAPB Wheat Ridge CO (2008).

[225] Meyerholz L Irzinger J Witthöft M Gerlach AL Pohl A. Contingent biofeedback outperforms other methods to enhance the accuracy of cardiac interoception: a comparison of short interventions. J Behav Ther Exp Psy. (2019) 63:12–20. 10.1016/j.jbtep.2018.12.002 30557753

[226] Schandry R Weitkunat R. Enhancement of heartbeat-related brain potentials through cardiac awareness training. Int J Neurosci. (1990) 53:243–53. 10.3109/00207459008986611 2265945

[227] DeCharms RC. Applications of real-time fMRI. Nat Rev Neurosci. (2008) 9:720–9. 10.1038/nrn2414 18714327

[228] Caria A Veit R Sitaram R Lotze M Weiskopf N Grodd W. Regulation of anterior insular cortex activity using real-time fMRI. Neuroimage. (2007) 35:1238–46. 10.1016/j.neuroimage.2007.01.018 17336094

[229] Veit R Singh V Sitaram R Caria A Rauss K Birbaumer N. Using real-time fMRI to learn voluntary regulation of the anterior insula in the presence of threat-related stimuli. Soc Cogn Affect Neur. (2011) 7:623–34. 10.1093/scan/nsr061 21983794 PMC3427870

[230] Zilverstand A Sorger B Sarkheil P Goebel R. fMRI neurofeedback facilitates anxiety regulation in females with spider phobia. Front Behav Neurosci. (2015) 9:148. 10.3389/fnbeh.2015.00148 26106309 PMC4458693

[231] Lawrence EJ Su L Barker GJ Medford N Dalton J Williams SC. Self-regulation of the anterior insula: reinforcement learning using real-time fMRI neurofeedback. Neuroimage. (2014) 88:113–24. 10.1016/j.neuroimage.2013.10.069 24231399

[232] Buyukturkoglu K Roettgers H Sommer J Rana M Dietzsch L Arikan EB. Self-regulation of anterior insula with real-time fMRI and its behavioral effects in obsessive-compulsive disorder: a feasibility study. PLoS ONE. (2015) 10:e0135872. 10.1371/journal.pone.0135872 26301829 PMC4547706

